# Structural Insight into the interaction of Flavonoids with Human Telomeric Sequence

**DOI:** 10.1038/srep17574

**Published:** 2015-12-02

**Authors:** Arpita Tawani, Amit Kumar

**Affiliations:** 1 Centre for Biosciences and Biomedical Engineering, Indian Institute of Technology Indore, Indore, Madhya Pradesh, India

## Abstract

Flavonoids are a group of naturally available compounds that are an attractive source for drug discovery. Their potential to act as anti-tumourigenic and anti-proliferative agents has been reported previously but is not yet fully understood. Targeting human telomeric G-quadruplex DNA could be one of the mechanisms by which these flavonoids exert anticancer activity. We have performed detailed biophysical studies for the interaction of four representative flavonoids, Luteolin, Quercetin, Rutin and Genistein, with the human telomeric G-quadruplex sequence tetramolecular d-(T_2_AG_3_T) (Tel7). In addition, we used NMR spectroscopy to derive the first model for the complex formed between Quercetin and G-quadruplex sequence. The model showed that Quercetin stabilises the G-quadruplex structure and does not open the G-tetrad. It interacts with the telomeric sequence through π-stacking at two sites: between T1pT2 and between G6pT7. Based on our findings, we suggest that Quercetin could be a potent candidate for targeting the telomere and thus, act as a potent anti-cancer agent.

The importance of human telomeric ends having the sequence d-(TTAGGG)_n_ is evident in somatic cells in which telomeres reach a critical limit (Hayflick limit), leading to the shortening of chromosomes and apoptosis^1^,^2^. In 80–85% of tumours, the aberrant cell proliferation and immortalisation is due to overexpression of a ribonucleozyme called telomerase, which maintains the length of telomeres by adding hexanucleotide repeats to the 3´ ends^3^,^4^,^5^. Telomerase utilises this end as primer for its reverse transcriptase (hTERT) activity, using its own hRNA component as a template for DNA synthesis^6^,^7^. Recent studies revealed that the G-quadruplex inhibits this enzymatic activity by dissociating the primer from the RNA template^8^,^9^. Therefore, the replication process of cancer cells via elongation of telomeric ends could be interrupted by stabilisation of the G-quadruplex, suggesting this mechanism as a potent cancer target for therapeutics^8^. Thus, the significance of the telomeric G-quadruplex in cancer treatment drives the exploration of small molecules that induce the formation of G-quadruplexes or that stabilise these structures^10^,^11^.

For the last decade, considerable research has been focused on exploiting small molecules^12^ with extended planar aromatic moieties, allowing them to intercalate into G-quadruplexes and form a stable conformation, but the major limitation to using these molecules is cytotoxicity and other side effects. Low molecular weight ligands with a lower level of systemic toxicity and high selectivity for the G-quadruplex could be effective telomerase inhibitors that induce the formation or stabilisation of the G-quadruplex^13^,^14^,^15^,^16^. Considering this, many groups have investigated the interaction of the G-quadruplex with naturally available small molecules such as berberine^17^, sanguinarine^18^, and others^19^,^20^,^21^, which usually offer relatively less toxicity and fewer side effects than the synthetic molecules. Most of these compounds stabilise the G-tetrad by π-stacking due to the presence of extended aromatic rings^22^. Among these, flavonoids are one of the important naturally available small molecules in our daily diets and have been considered for use as potential drug candidates for anticancer therapy^23^. The common dietary flavonoids Luteolin, Quercetin, Rutin and Genistein have received significant attention for their protective as well as potentially destructive anti-tumour, anti-cancer and anti-oxidant activities^23^,^24^,^25^,^26^,^27^,^28^. Various mechanisms have been proposed for such activities of these flavonoids involving the inhibition of phosphatidylinositol 3-kinase, phosphorylase kinase and DNA topoisomerases^29^,^30^. Furthermore, flavonoids have been reported to exert their anti-cancer effects at different stages of cancer development and inhibit cellular proliferation, induce cellular cytotoxicity by modulating mitogenic and apoptotic signalling pathways, and affect cell-cycle regulation, among other activities^31^. The anti-proliferative and cytotoxic effects of these flavonoids on different cancer cell lines have also been well studied, and Quercetin exhibited the lowest EC_50_^32^,^33^.

Despite many studies on the anti-tumour, anti-proliferative and anti-apoptotic activities of these flavonoids, the major cellular target of their action remains elusive. Moreover, it has been known that ligands with planar aromatic regions intercalate effectively into the G-quadruplex structure and stabilise it^34^. The flavonoid skeleton contains a planar chromophore with an additional carboxyl group for protonation and can effectively intercalate into the planar scaffold of G-tetrads^35^,^36^.

Previous studies suggest that among the major known flavonoids, the interaction of these flavonoids with nucleic acid (calf thymus) was on the order of Quercetin > Kaempferol > Delphinidin, with K_que_ = 7.25×10^4^ M^–1^^37^. Furthermore, studies on interactions of flavonoids with triplexes and tetraplexes showed that Quercetin interacts favourably with tetraplexes with a *K*_ass_ value of ~10^3^ M^–1^ for human telomeric sequences^38^. The reported anticancer effects of Quercetin, the most intensely studied flavonoid, prompted us to investigate its mode of interaction with the human telomeric G-quadruplex DNA sequence.

Previously, spectroscopic studies have been performed to investigate the interaction of Quercetin with monomeric and dimeric G-quadruplexes. The results suggest that Quercetin acts as a groove binder for the monomeric conformation, whereas it binds to the dimeric conformation via a stacking mode^39^. Although extensive research has examined the interaction of flavonoids with nucleic acids^37^,^38^, no structural studies assessing their interaction with the human telomeric G-quadruplex sequence has been reported in the literature to date.

Herein, we report the first structure for the flavonoid Quercetin complexed with Tel7 G-quadruplex sequence d-(TTAGGGT). Our present study focused on four different flavonoids, Luteolin, Quercetin, Rutin and Genistein, and their interaction with Tel7 G-quadruplex sequence. NMR studies along with other biophysical techniques, such as Circular Dichroism (CD), visible absorption and steady-state and time-resolved fluorescence spectroscopies, were employed to investigate the binding mode of these flavonoids to Tel7 G-quadruplex DNA. Furthermore, this study aimed to achieve a structural basis for the interaction and stabilisation of the intermolecular parallel G-quadruplex DNA by the most abundant naturally occurring flavonoid^40^. Our study revealed that all of the flavonoids bind to Tel7 G-quadruplex DNA. Furthermore, detailed structural studies revealed that Quercetin binds to Tel7 G-quadruplex DNA via intercalation between the T1pT2 and G6pT7 steps.

## Results

**Figure 1:**
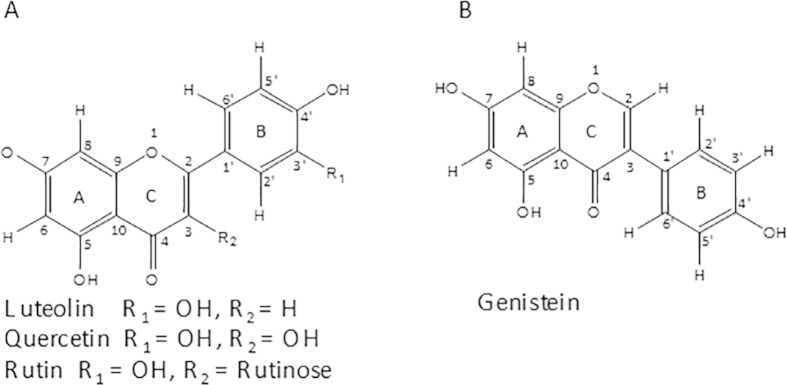
Structure of flavonoids. (**A**) Luteolin, Quercetin, Rutin (**B**) Genistein.

In this study, the details of the interaction between flavonoids and Tel7 G-quadruplex DNA were explored. Flavonoids (Fig. 1) are naturally occurring molecules and omnipresent in fruits and vegetables. The binding of flavonoids with human telomeric G-quadruplex DNA was explored using various biophysical techniques and shown to stabilise the G-quadruplex structure.

### Exploration of flavonoid binding to Tel7 G-quadruplex DNA by absorbance and fluorescence spectroscopy

Electronic absorption spectroscopy is one of the useful techniques for DNA-binding studies^41^. All four flavonoids exhibited two absorption bands, one near the visible region (340 nm–370 nm), attributed to the conjugated benzopyran ring, and the other in the ultraviolet region, attributed to the aromatic phenyl ring of these flavonoids^42^. Luteolin and Genistein exhibited an absorption band near the visible region at 350 nm, whereas Quercetin and Rutin showed absorption bands at 368 nm and 365 nm, respectively. Upon titrating Tel7 G-quadruplex DNA into these flavonoids, the near to visible bands showed a red shift of 11 nm, 15 nm and 3 nm for Luteolin, Quercetin and Rutin, respectively, whereas Genistein showed a blue shift of 2 nm. A 9–29% hyperchroism was also observed for all of the four flavonoids (see Supplementary Figure S3 and Table S1). Such changes in spectral profile are consistent with previous reports and indicated formation of the G-quadruplex- flavonoid complex^39^.

The binding affinities of flavonoids to Tel7 G-quadruplex DNA were monitored by means of fluorescence titration experiments. The fluorescence emission of all four flavonoids was examined at the emission maximum in their unbound form. The fluorescence emission of these flavonoids was very weak, most likely due to the torsional motion of the phenyl and c-pyrone rings. On addition of Tel7 G-quadruplex DNA to the flavonoid solution, an enhancement in the fluorescence intensity was observed for Luteolin, Quercetin and Rutin, while quenching was observed for Genistein. These observed spectral changes depicted the binding of these flavonoids to the G-quadruplex DNA and generated the G-quadruplex-flavonoid complex. The binding curve was obtained by fitting the plot of ΔF (change in the fluorescence intensity) against the G-quadruplex DNA concentration with a ligand binding two site saturation model (see Supplementary Figure S4). The binding constant values (K_d_) of these flavonoids (as listed in the Table 1a) strongly suggested a higher affinity of Quercetin compared to the other flavonoids. In order to understand the binding behaviour of Quercetin with other biologically relevant G-quadruplex DNA, we have also performed the fluorescence titration experiment of Quercetin with Tel22, c-myc and c-kit G-quadruplex DNA. The binding constant values of Quercetin (as listed in the Table 1b) obtained from this experiment suggested that Quercetin binds to G-quadruplex DNA sequences (see Supplementary Figure S6). The mode of binding as observed are slightly different and that could be due to the difference topologies of G-quadruplex structures formed by different G-rich DNA^43^. Further, in order to determine the specific binding of these flavonoids, the fluorescence titration experiment was also performed with ct-DNA (see Supplementary Figure S5). The binding constant values of these flavonoids with ct-DNA were lower as compared to Tel7 G-quadruplex DNA. Amongst which Quercetin shows ~1000 fold higher affinity for Tel7 G-quadruplex DNA. These results suggested the high specificity and affinity of Quercetin for various G-quadruplex forming DNA sequences viz. Tel7, Tel22, c-myc and c-kit (Table 1b and Supplementary Figure S6).

### Formation of the G-quadruplex-flavonoid complex studied using time resolved fluorescence measurements

**Figure 2:**
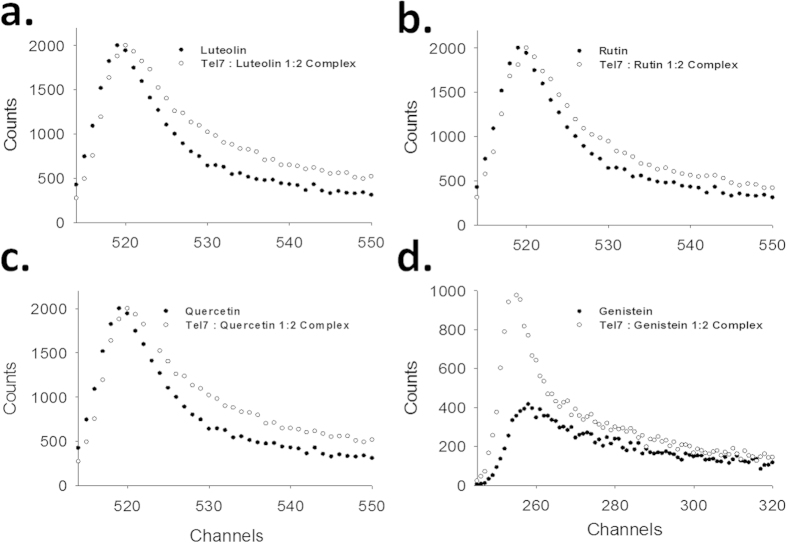
Fluorescence lifetime decay curve at 2:1 D/N ratio for (a) Luteolin (b) Quercetin (c) Rutin (d) Genistein.

In aqueous solution, the changes in the photophysical process of an excited fluorescent probe can be inferred via time-resolved fluorescence decay studies. As fluorescence excited state lifetimes are very sensitive to the structure and dynamics of a fluorophore, this study provides insight into how DNA structure influences the fluorescence decay profiles of these flavonoids. The fluorescence decay of free flavonoids is triexponential and attributed to the presence of three different conformations in the solution, which were likely due to the rotation of a single bond between benzopyran rings to phenyl rings. Thus, the decay profiles of free flavonoids have three lifetimes (τ1, τ2 and τ3) and three amplitudes (β1, β2 and β3) (see Supplementary Table S2a). The binding sites of flavonoids on the Tel7 G-quadruplex DNA were inferred by comparing their decay profile in the free state to that in 2:1 molar ratio of the Drug/Nucleic acid (D/N). From Time-Correlated Single Photon Counting (TCSPC) analysis, it was observed that the fluorescence decay lifetime of the free flavonoids was decreased compared to that of the G-quadruplex-flavonoid complex (Fig. 2). Upon addition of Tel7 G-quadruplex DNA, Genistein showed the lowest decay rate (in the range of ps) with the highest amplitude, which may be attributed to the single binding pattern of Genistein. Other flavonoids followed the trend of shorter and longer lifetimes for free and complexed flavonoids, respectively, along with slight changes in amplitudes. This stipulated the presence of a different pattern of complex formation, attributed to the binding of flavonoids to the G-quadruplex structure at more than one site.

The binding data from fluorescence titration experiment clearly suggested that the binding of Quercetin to Tel22 was more or less similar to that of Tel7, thus we have also performed TCSPC experiment of Quercetin with Tel22 (see Supplementary Figure S7). The life time decay profile of free Quercetin was decreased as compared to that of its complex with Tel22 DNA in D/N = 2:1 (see Supplementary Table S2b). This also confirms the binding of Quercetin to Tel22 G-quadrupelx DNA.

### Insight into the interaction of Tel7 G-quadruplex DNA – flavonoid complex using NMR spectroscopy

**Figure 3:**
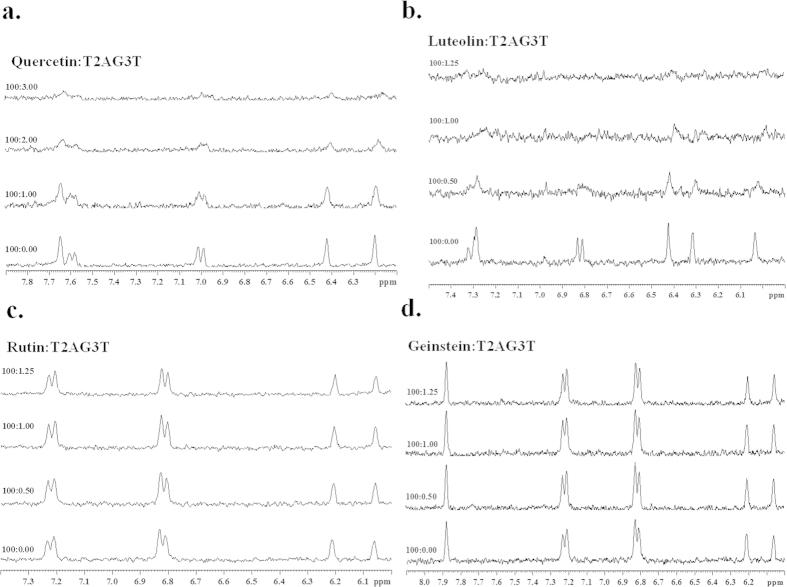
One dimensional proton spectra for flavonoids and Tel7 complex. NMR titration of 200 μM of flavonoids with increasing concentration of Tel7 (**a**) Luteolin (**b**) Quercetin (**c**) Rutin (**d**) Genistein.

NMR titration of flavonoids upon addition of Tel7 G-quadruplex DNA was performed. The change in the shape of the resonances of flavonoid protons were observed with the successive addition of DNA to the flavonoid solution (Fig. 3). At D/N ratio of 100:1, the broadening of the Quercetin and Luteolin proton were seen, while Rutin and Genistein protons showed no changes even at higher concentration of DNA. These broadening of signals were accompanied by their disappearance at D/N ratio of 100:1 for Luteolin and 100:3 for Quercetin. This result also suggests the involvement of these protons in the binding of flavonoids with Tel7 G-quadruplex DNA.

However, the data from the other biophysical experiments performed in this study suggested that the Quercetin showed the highest affinity for the Tel7 G-quadruplex DNA amongst all four flavonoids used in this study. Therefore, we performed the detailed NMR studies with Quercetin in order to understand the structural basis of its interaction with Tel7 G-quadruplex DNA.

### Understanding the structure of Tel7 G-quadruplex DNA - Quercetin complex using NMR spectroscopy and restrained Molecular Dynamic (rMD) simulation

**Figure 4:**
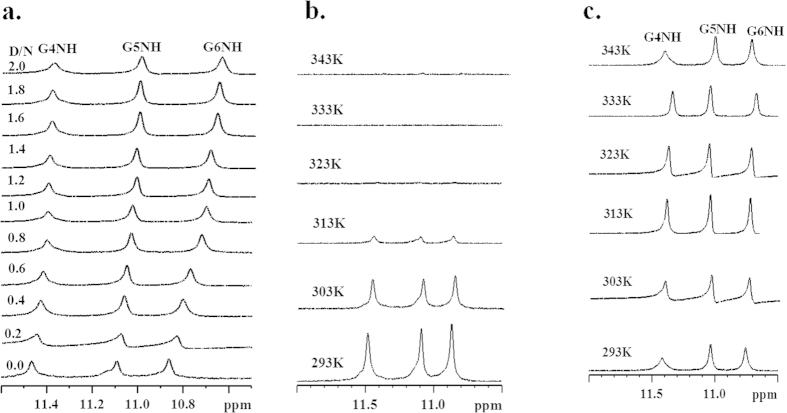
NMR spectra for Quercetin and Tel7 complex. (**a**) ^1^H NMR spectra showing interaction of Quercetin with Tel7 monitored by imino region as a function of ligand/DNA ratio at 298 K. (**b**) ^1^H NMR spectra of Tel7 monitored by imino region at different temperature. (**c**) ^1^H NMR spectra showing interaction of Quercetin with Tel7 monitored by imino region as a function of temperature ligand/DNA ratio = 2.0.

**Figure 5:**
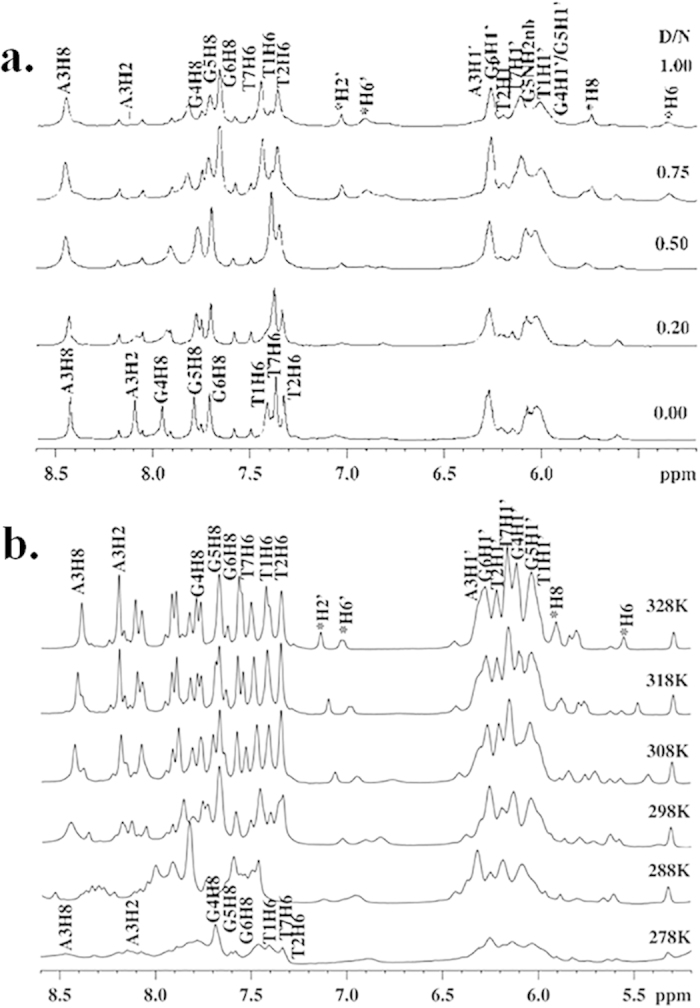
One dimensional proton spectra for Quercetin and d-(T_2_AG_3_T)_4_ complex. (**a**) Interaction of Quercetin with Tel7 monitored by base proton and H1′ region as a function of increasing concentration of Quercetin upto D/N = 1.0 ratio at 298 K. (**b**) Interaction of Quercetin with Tel7 monitored by base proton region as a function of temperature at D/N = 1.0 ratio. Proton resonances from Quercetin are marked with asterisk.

NMR experiments were performed to elucidate the binding sites of Quercetin with Tel7 G-quadruplex DNA. The NMR spectra of the Tel7 forming the G-quadruplex structure was depicted by three well-resolved resonances in the imino region (10–12 ppm) of ^1^H-NMR. Unambiguous assignment of all resonances was performed on the basis of a previously reported strategy^44^. Most likely due to the solvent exchange, the peaks for the thymine imino protons were not observed. In the ^1^H-NMR spectra of unbound G-quadruplex DNA, 11.45, 11.10 and 10.86 ppm mark the resonances for G4NH, G5NH and G6NH, respectively (Fig. 4a, D/N = 0.0). We have performed NMR titration experiments to characterise the interaction of Quercetin with the Tel7 sequence. As Quercetin was gradually titrated into the Tel7 G-quadruplex DNA solution, remarkable changes in chemical shifts were observed in imino (Fig. 4a) as well as other regions of the proton NMR spectrum (Fig. 5a and SI Figure S8a,b). The upfield shift of G6NH, G5NH, G4NH resonances of Tel7 upon the addition of Quercetin are shown in Fig. 4a. Along with this upfield shift, the slight downfield shift of the T1H6, T2H6 and T7H6 resonances were also observed. These changes in chemical shift were accompanied by significant changes in the shape of resonances as more Quercetin was added to the Tel7 G-quadruplex DNA solution. After D/N = 1.0, the remarkable broadening of the G4NH, T7H6, G6H8, A3H2, T1H6, and T2H6 peaks suggested that the binding site of Quercetin on Tel7 was most likely close to the T1/T2/A3 or G6/T7 base step (see Supplementary Figure S8a,b). In the imino region at D/N = 2.0, G6NH resonance showed the largest change in chemical shift value of ~0.20 ppm (Fig. 4a). These upfield shifts for the imino resonances are in line with previously reported results^39^ and could be due to its π-electronic cloud^45^, suggesting the stacking of Quercetin below the G6-tetrad.

The binding of Quercetin to the Tel7 G-quadruplex DNA was also assessed using temperature-dependent NMR studies. The rise in temperature causes the breaking of hydrogen bond in G-quartet structure, which results in the disappearance of imino proton signals. At D/N = 0.0, the imino proton resonances start disappearing at 313 K and were completely lost at 323 K. However, at D/N = 2.0, these imino proton resonances can be seen upto 343 K. These results clearly show that binding of Quercetin stabilizes the G-quadruplex structure (Fig. 4b,c).

Moreover, at D/N = 2.0, with the increase in temperature, the resonances of Quercetin H6, H8, H2´ and H6´ protons become sharp and clearly visible, which were otherwise slightly broader at low temperature. Additionally, the downfield shift in the Quercetin proton resonance was observed in only the complex, but no such shift is observed for free Quercetin, suggesting the involvement of these protons in binding (Fig. 5b)^44^.

**Figure 6:**
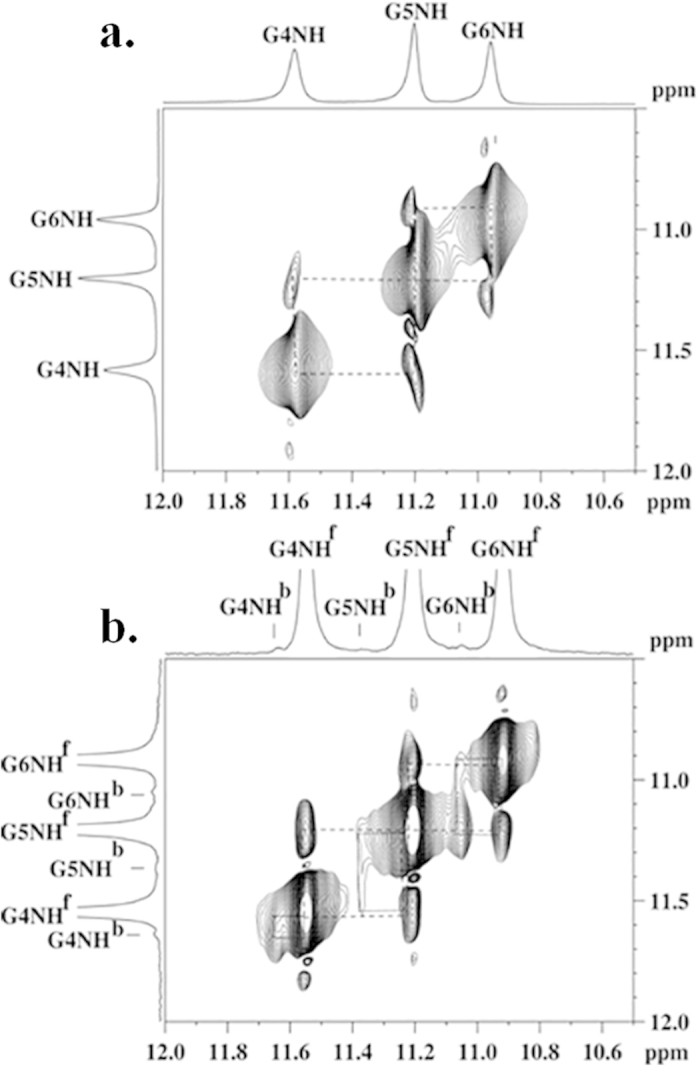
Portion of 200ms NOESY spectrum of Tel7- Quercetin complex. (**a**) Portion of 200 ms NOESY spectrum of Tel7 - Quercetin complex showing NH-NH NOEs between adjacent G-tetrads at 298 K at ligand/DNA ratio = 1.0. (**b**) Free and bound NH signals due to Drug-DNA complex formation at 318 K at ligand/DNA ratio = 1.0.

NOESY spectra were collected at different mixing times and at three different temperatures (288 K, 298 K and 318 K) for the Quercetin-Tel7 complex at D/N = 0.25, 0.50, 0.75, 1.00, 1,5 and 2.00. Upon addition of Quercetin to Tel7 G-quadruplex DNA, the emergence of a new cross peak for G5NH and G6H8 was observed. As observed in the imino region of the NOESY spectra, the emergence of new sets of cross peak at 318 K indicated the presence of free DNA and Drug-DNA complex in solution (Fig. 6). The unbound form of Tel7 displayed strong NOEs accounted by intra-nucleotide connectivity and sequential connectivity, including those of the imino protons (Fig. 6a and SI Figure S9a). These strong NOEs denoted a well-established stacking interaction between DNA base pairs.

As Quercetin was titrated into the Tel7 solution, few of the existing NOEs disappeared and some new NOEs emerged. The loss of the cross peak at 11.67 ppm for G4NH with A3H2 (see Supplementary Figure S9b) and the emergence of a new cross peak for G5NH and G6H8 suggested the binding of two Quercetin molecules, one near the A3 residue and other near the G6 residue. Additionally, in the Drug-DNA complex, it was observed that the NOEs between the A3-G4 base became weak (see Supplementary Figure S9b). This accounts for the perturbation that occurs in the stacking interactions as Quercetin intercalates at the T1pT2 step.

**Figure 7:**
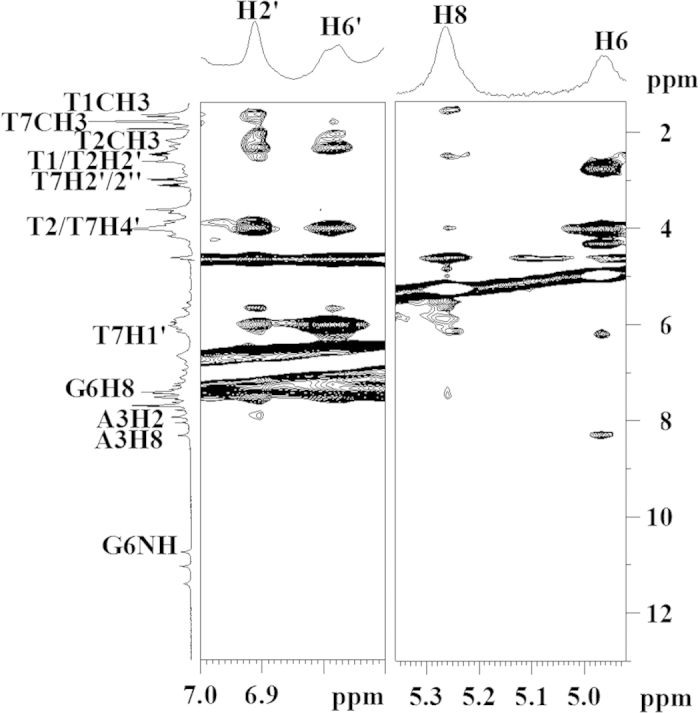
Portion of 200ms NOESY spectrum of Tel7 - Quercetin complex. NOESY spectrum at D/N = 2.0 showing intramolecular cross peak within Quercetin and intermolecular cross peaks of Quercetin H2´, H6´ and Quercetin H8, H6 with Tel7.

**Figure 8:**
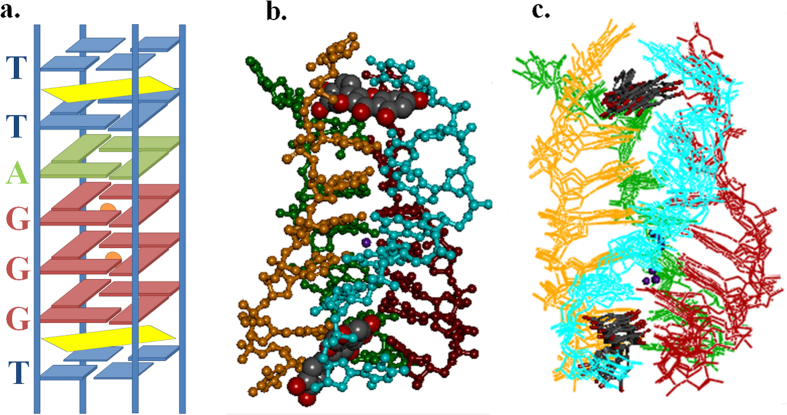
Quercetin and Tel7 complex at D/N = 2.0. (**a**) Schematic representation showing Quercetin (yellow) stacking at the T1pT2 and G6pT7 steps. Potassium ions (orange) are bound at the GpG steps (PDB Code: 2MS6). (**b**) Energy minimised model of Quercetin and Tel7 complex showing Quercetin intercalates at the T1pT2 and G6pT7 steps. All the four strands of Tel7 are shown in red, green, cyan and magenta. (**c**) Ensemble of five lowest energy structures after restrained molecular dynamics simulation showing the position of Quercetin at the T1pT2 (top) and G6pT7 steps (bottom). All the four strands of d-(T_2_AG_3_T)_4_ are shown in red, green, cyan and orange.

We have performed NMR titration experiment of Quercetin to determine which Quercetin protons were involved in the interaction with Tel7 G-quadruplex DNA. As the concentration of Tel7 G-quadruplex DNA increases, the Quercetin proton signals broadened and disappeared at D/N ratio of 100:3 (Fig. 3a). Furthermore, 24 NOEs were observed for the H6, H8, H2´ and H6´ Quercetin protons with the base and sugar protons of the T1, T2 and T7 residues (Fig. 7), suggesting the position of the Quercetin benzopyran ring in between the bases. This is further confirmed by the NOEs obtained between Quercetin H2´ to T7H1´ and T1/T2 H2´/H2´´ (Fig. 7), showing the end stacking mode of ligand binding to DNA. Briefly, all of the results indicate the binding of Quercetin to two sites on Tel7 by intercalating between the T1pT2 and G6pT7 steps via the end stacking mode (Fig. 8a).

To gain a better understanding of the Quercetin-G-quadruplex interaction, restrained molecular dynamics (rMD) simulation was performed using Discovery Studio Client 3.5 (Accelrys, San Diego, CA). Quercetin was docked at the T1-T2 step and the G6-T7 step in an orientation that satisfied all of the NOE restraints. The cross peak intensities were used in a qualitative manner, in which the distances were approximately 1.8–2.5, 2.5–3.0, 3.0–3.5, 3.5–4.0, and 4.0–5.0 Å for strong intense (ss), strong (s) medium (m) and weak (w) and very weak intense (vw) peaks, respectively (see Supplementary Table S5). After the production runs of 1 ns, an ensemble of five conformations with the lowest potential energy were superimposed (Fig. 8c).

The rMD simulation results showed that one of the Quercetin molecules stacks below the G6 tetrad and the other intercalates at the T1-T2 step (see Supplementary Figure S11), which complies with the previous conclusions obtained from the other biophysical experiments performed. The energy minimised model for the Quercetin-Tel7 complex obtained after restrained molecular dynamic simulations (Fig. 8b) has final potential energy of −10256.458-kcal/mol (see Supplementary Table S3, S4). The results favoured the distances obtained from the NOE experiment and show that Quercetin acts as an intercalator, binding to Tel7 between the T1pT2 and G6pT7 position of the G-quadruplex (see Supplementary Table S5).

## Discussion

It is well known fact that flavonoids have potent anti-cancer activity that is likely involved in their cellular binding targets. The human telomeric G-quadruplex DNA Tel7 sequence could be one of the binding targets of flavonoids that generate anti-cancer activity. Thus, the examination of the binding of flavonoids to the human telomeric DNA is important. In the present study, various biophysical experiments were performed to gain insight about the interaction of flavonoids with the human telomeric G-quadruplex DNA Tel7 sequence.

Prior to discussing the binding mode and revealing the binding sites of flavonoids on the Tel7 DNA, it is necessary to explain the structural conformation of the Tel7 G-quadruplex DNA in K^+^ solution. CD spectroscopy is often the ideal technique to recognise the conformation of DNA. The Tel7 sequence is known to form a parallel G-quadruplex structure in K^+^ solution^44^, which is evident by a positive peak at 260 nm and a trough at 240 nm, as exhibited in the CD spectra for Tel7 sequence (see Supplementary Figure S1). This is further supported by the ^1^H-NMR spectra of the unbound Tel7 sequence. The presence of clear peaks in the 10–12 ppm region of the ^1^H-NMR spectra is a unique feature of higher ordered DNA structures such as G-quadruplexes. Thus, the three well-resolved imino proton signals in the 10–12 ppm region confirm the formation of the G-quadruplex structure by Tel7 in K^+^ solution.

The verification of the binding of flavonoids to Tel7 can be assessed from absorption and fluorescence titration experiments. The absorption spectra are one of the common methods to determine drug binding to DNA. The observed spectral changes in absorption titration, including the hyperchroism observed for all of the flavonoids and the red shift observed in case of all flavonoids except Genistein, confirms the binding of the flavonoids to the G-quadruplex DNA. Concomitant with this observation, a significant enhancement in fluorescence intensity also suggested the binding of flavonoids to the Tel7 G-quadruplex DNA. As can be clearly observed from the fluorescence titration data for the flavonoids and G-quadruplex DNA, Quercetin showed the highest affinity for the Tel7 G-quadruplex DNA among all of the flavonoids used in this study. The fluorescence titration data of flavonoids with ct-DNA and Tel22 G-quadruplex DNA also confirms the specific binding of flavonoids to any kind of G-Quadruplex DNA sequences. Along with this specificity, Quercetin is found to be more selective to G-quadruplex formed by telomeric DNA sequences as compare to other G-quadruplex forming DNA sequences like c-myc and c-kit promoter sequences (Figure S6 and Table 1b). This could be due to the difference in topologies of G-quadruplex that depends upon its sequence, strand directionality, its combination, loop size and its sequence^46^. Also, the sequence selectivity of Quercetin might be the reason for observing binding constants in relative manner with binding constants in the order of Tel22 > cmyc > ckit. Thus, to gain insight about the binding of Quercetin and G-quadruplex DNA with more accuracy, proton NMR spectroscopy was employed. NMR is one of the most sensitive techniques to explore ligand-DNA interactions. NMR titration experiments were performed to verify the binding of Quercetin to Tel7 G-quadruplex DNA. With the addition of Quercetin to the DNA solution, significant changes in the chemical shifts and the broadening of peaks were observed. This observation was consistent with changes observed in the temperature-dependent NMR experiments. At D/N = 1:1 ratio, an increase in temperature caused the emergence of a new set of peaks upfield of the original resonances, indicating the binding of Quercetin to the G-quadruplex DNA. The co-existence of resonances for free DNA and the drug-DNA complex in the solution (Fig. 6b) stipulated that binding takes place in slow exchange regime on the NMR time scale^47^ and is usually inferred as specific and tight binding^48^.

Once the binding of Quercetin to DNA was confirmed, the next step was to elucidate the binding site and the mode of binding to Tel7. The binding site of Quercetin to the Tel7 G-quadruplex DNA could be obtained from 1D/2D NMR experiments. In the ^1^H NMR spectra of the unbound G-quadruplex sequence, the well-defined signals in the imino region and the aromatic region enabled us to determine the binding sites of Quercetin on the G-quadruplex DNA. A change in chemical shifts was observed as more Quercetin was added to the G-quadruplex DNA. As it is clearly observed in Fig. 4a, the binding of Quercetin to the DNA causes perturbations in the chemical shifts of the imino resonance of guanine residues and the H6 resonances of other residues (Fig. 5a and SI Figure S8a,b). The largest perturbation was observed for the G6 imino resonance, which was shifted upfield. A slight downfield shift of the H6 proton resonance was also observed for the T1, T2 and T7 residues. Moreover, binding site localisation could also be concluded from dramatic changes observed in temperature-dependent NMR studies. At lower temperatures the peaks were broadened significantly but sharpened as temperature rose, with the resonance of the G6 imino proton being the most affected (Fig. 4c). All of the above observations strongly suggested the binding of Quercetin near the T1/T2 or G6/T7 base step.

The binding mode of the ligand to the DNA could be obtained by analysing the fluorescence lifetime decay profile using TCSPC studies. Usually, planar molecules with an extended aromatic core interact with the G-quadruplex structure via two modes: either via intercalation or via end-stacking and external or groove binding. The lifetimes in the end-stacking mode are larger than those in the external binding^49^. Additionally, previous reports showed that Quercetin interacts with G-quadruplexes by end-stacking and outside binding^39^. Thus, the significant changes observed in the values of both the decay components and the amplitudes of the flavonoids upon binding to Tel7 at D/N = 2:1 ratio clearly indicate the intercalation of flavonoids with Tel7 and the formation of the complex. This intercalation mode could also be supported by the observation made from the NOESY experiments. The loss of sequential connectivity between T1H1´-T2H6 in the drug-DNA complex in the NOESY spectra occurred due to the intercalation of the Quercetin chromophore at these base steps (see Supplementary Figure S10). The loss of the G4NH and A3H2 cross peak and the emergence of new NOEs supported the binding of two molecules of Quercetin to the Tel7 G-quadruplex DNA near the T1/T2 or G6/T7 base step, which was also observed in the ^1^H NMR spectra as stated above.

The Quercetin molecule also orients itself in such a way that it achieved better interaction with the T1/T2 or the G6/T7 base step in the intercalative mode, with the H6, H8, H2´ and H6´ protons involved in the binding. The participation of Quercetin H6, H8, H2´ and H6´ protons in binding was corroborated by the clear visibility of these protons at high temperature, which occurred due to the weakening of the Drug-DNA interactions and results in destacking (Fig. 5b and SI Figure S8c). Further, with the successive addition of Tel7 G-quadruplex DNA to the Quercetin solution, the broadening and finally disappearance of these proton signals also suggested their involvement in the binding with Tel7 G-quadruplex DNA (Fig. 3a). Moreover, we have also performed rMD studies to collect detailed conformational analysis. The rMD simulations were based on the inter-molecular and intra-molecular NOEs obtained in the NOESY experiments. The results of the rMD simulation also suggested the intercalation of the Quercetin chromophore at the T1/T2 or G6/T7 base step, which was stabilised by the stacking interactions (Fig. 8).

The NMR melting experiment showed that the binding of Quercetin to the Tel7 sequence stabilises the G-quadruplex structure (Fig. 4b). It is well known that generally G-quadruplex ligands have an extended aromatic plane. Quercetin provides the same molecular frame for π- stacking and might stabilise the G- quadruplex structure by the strong π–π stacking between guanine tetrads and the Quercetin chromophore. The loss of inter-nucleotide NOEs was also not observed for the core G-tetrad. Additionally, the existence of intra-nucleotide sequential connectivity along with the appearance of new NOEs in the NOESY spectra (Fig. 6) prove that the G-tetrad is intact and does not open to provide access to Quercetin. This result is also supported by our CD experiment in which we observed no changes when Quercetin and other flavonoids were bound to DNA, even at D/N = 2:1 ratio (see Supplementary Figure S1). Further, we have also performed this experiment for Quercetin with Tel22 which is biological relevant sequence of Tel7. The CD data for Tel22 and Quercetin shows mode of interaction similar to Tel7, confirming that Quercetin stabilises the G-quadruplex structure of Tel7 or Tel22 in similar fashion (see Supplementary Figure S1 and Figure S2). Furthermore, the stabilization of G-quadruplex structure was also evident by PCR stop assay. The observed decrease in the intensity of the PCR product with increasing concentration of Quercetin (lost of band at 12.5 μM) indicates that binding of Quercetin stabilize the G-quadruplex (Tel22) and blocks *Taq*polymerase activity of DNA amplification (see Supplementary Figure S12). All of these results strongly suggest that the Quercetin does not hamper the G-tetrad structure, but rather it stabilises the G-quadruplex structure by binding in the intercalation mode to neighbouring bases.

In conclusion, we reported the interaction of four flavonoids (Luteolin, Quercetin, Rutin and Genistein) with the human telomeric Tel7 G-quadruplex structure formed by the Tel7 sequence and the stabilisation of this structure. The binding properties of flavonoids with the G-quadruplex structure have been characterised via CD spectroscopy, UV-Vis and fluorescence titration studies. The mode of binding was deduced according to analysis of their lifetime decay profiles. Quercetin bound the most effectively according to the binding studies performed. Thus, a better understanding of the interaction between Quercetin and Tel7 was deduced from the resolved model. This interaction occurs via π- stacking in D/N = 2:1 ratio, and Quercetin intercalates at the T1pT2 and G6pT7 steps. Our investigation highlights the structural aspects of the binding of flavonoids to G-quadruplexes formed by the human telomeric DNA sequence and also revealed the potential of flavonoids as useful candidates for anti-cancer therapeutics by regulating the telomeric G-quadruplex structure. The coordinates of the NMR model of the Quercetin and Tel7 complex have been deposited in the PDB as 2MS6.

## Methods

### Reagents

Luteolin, Quercetin, Rutin and Genistein were purchased from Sigma Aldrich Chemicals Ltd. These flavonoids were used without further purification. The solvents, including deuterium oxide, dimethyl sulphoxide (DMSO) and other reagents used for buffer preparation such as NaCl, KCl, NaH_2_PO_4_, Na_2_HPO_4_, KH_2_PO_4_ and K_2_HPO_4_ (HPLC Grade) were also purchased from Sigma Aldrich Chemicals Ltd. The stock solutions of flavonoids were prepared by dissolving them in DMSO, and they were stored at the appropriate storage temperature. The concentration of the solutions was determined spectrophotometrically at the λ_max_ of 380 nm (ε = 14,920 M^−1^ cm^−1^), 368 nm (ε = 19,700 M^−1^cm^−1^), 349 nm (ε = 20,417 M^−1^cm^−1^) and 260 nm (ε = 37,260 M^−1^cm^−1^) for Quercetin, Rutin, Luteolin and Genistein, respectively.

Calf thymus DNA (ct-DNA) and other DNA oligomers i.e. Tel7: d-(5′-TTAGGGT-3′), Tel22: d-(5′-AGGGTTAGGGTTAGGGTTAGGG-3′), c-myc: d-(5′**-** TGAGGGTGGTGA GGGTGGGGAAGG-3′), ckit21up: d-(5′**-** CGGGCGGGCGCGAGGGAGGGG-3′) were purchased from Sigma Aldrich Chemicals Ltd. ct-DNA solution was prepared in the sodium phosphate buffer and its concentration was measured spectrophotometrically using a molar absorptivity of 6600 M^−1^ cm^−1^ (260 nm).

For quadruplex formation, 100 μM of oligomers were dissolved in phosphate buffer (K^+^) (10 mM, pH 7.0) with 100 mM KCl. The oligomer was annealed by heating at 90 °C for 5 mins, followed by overnight incubation at room temperature to allow gradual cooling. The synthetic oligonucleotide sequence d-(T_2_AG_3_T) was purchase from Sigma Aldrich Chemicals Ltd.

### Absorbance Titrations and Fluorescence Titrations

The UV-Vis spectra for flavonoids and G-quadruplex-flavonoid complexes were obtained using Varian Cary 100 Bio UV-Visible Spectrophotometer. The spectra were recorded by progressive addition of G-quadruplex DNA to the fixed concentration of flavonoid solution and scanned at 25 ± 0.5 °C using a 10 mm (1 mL) quartz cell.

The fluorescence titration experiment was performed on Synergy™ H1 multi-mode microplate reader using 96-well microplates from corning. The volume of 75 μL for each sample was tested in duplicates at 25 °C. The G-quadruplex DNA at a final concentration of 10 μM was serially diluted; with the last well serve as blank (no DNA). The ct-DNA at a final concentration of 50 μM for Luteolin, Quercetin and Genistein, while 30 μM for Rutin was serially diluted; with the last well serve as blank (no DNA). The readings were taken at emission wavelength of 435 m, 535 nm, 416 nm and 405 nm for Luteolin, Quercetin, Rutin and Genistein respectively, when excited at the excitation wavelength of 380 nm, 375 nm, 360 nm and 269 nm. Data were analyzed using SigmaPlot 12.0 software (Systat Software, Chicago, USA) according to:


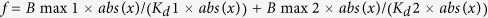


B_max_ = maximum number of binding sites.

K_d_ = equilibrium binding constant.

### Circular Dichroism

The Circular Dichroism (CD) experiment was performed on a J-815 Spectropolarimeter (JASCO). A constant temperature of 298 K was maintained during the entire experiment with the help of a Peltier junction temperature controller. To avoid water condensation on the outside of the cuvette, a constant stream of dry nitrogen gas was flushed into the cuvette-holding chamber. A quartz cuvette with a 0.2 cm path length was used to record the spectra of samples containing 20 μM G-quadruplex and increasing concentrations of flavonoids in 100 mM KCl, 10 mM phosphate buffer (K^+^) at pH 7.0. Spectra were recorded at 0.1 nm intervals from 200 nm to 350 nm with a 1 nm-slit width and averaged over three scans. Buffer CD spectra were subtracted from the CD spectra of DNA and the Drug-DNA complex.

### Time-resolved fluorescence measurements

The time-correlated single-photon counting (TCSPC) method was used to perform the lifetime measurement study. Time resolved fluorescence decays were collected on a Time-Correlated Single-Photon Counting (TCSPC) Spectrofluorometer (Horiba). A fixed wavelength Nano LED was used as the excitation source (k_ex_ = 470 nm), and emission was detected at a different wavelength. The fluorescence emission of the flavonoids and their complexes with G-quadruplex DNA were counted with a micro channel plate photo multiplier tube after passing through the monochromator and were further processed through a constant fraction discriminator (CFD), a time-to-amplitude converter (TAC) and a multi-channel analyser (MCA). The fluorescence decay was obtained and further analysed using DAS software, provided by FluoroLog-TCSPC instruments.

### Nuclear Magnetic Resonance

NMR experiments were conducted on a high-resolution AVANCE III 400 and 500 MHz BioSpin International AG, Switzerland equipped with a 5 mm broad band inverse probe able to deliver z-field gradients. NMR data were processed, integrated and analysed on Topspin (1.3 version) software. NMR samples were referenced with 3 - (Trimethylsilyl) propionic-2, 2, 3, 3-d_4_ acid sodium salt (TSP). The drug-DNA complex was formed by titrating Tel7 with successive additions of the drug. All of the titration studies were performed in H_2_O + D_2_O solvent at a 9:1 ratio. The 64K data points were recorded for 1D proton NMR spectra with 64–128 numbers of scan at 298K and a digital resolution of 0.15 - 0.3 Hz/point. To attain the best signal to noise ratio, the receiver gain was optimised at each instance. Two-dimensional proton nuclear overhouser enhancement spectroscopy (NOESY)^50^ experiments were performed at a temperature range of 298 K with 20 ppm spectral width. Spectra were recorded at variable mixing times (t_m_) of 350 ms, 300 ms and 200 ms with 256 free induction decays along the t_1_ dimension and 2048 complex data points in the t_2_ dimension. A digital resolution of 1.495 Hz/point in the t_1_ dimension was obtained in 48–64 scans with a relaxation decay of 2.0 secs. The titration studies for D/N = 0.00 , 0.20 upto 2.00 complex were performed at 100, 200 and 300 ms mixing time. SPARKY was used to visualise the spectra and calculate ^1^H-^1^H NOE distances, which were used to restrain Quercetin-Tel7 G-quadruplex DNA for restrained molecular dynamic simulation studies.

### Restrained Molecular Dynamics studies

The structure of G-quadruplex d-(T_2_AG_3_T)4 (PDB code: 1NP9)^44^ was taken as the starting model for the rMD studies. The required replacements, addition of residues and the G-quadruplex-Quercetin complex were built in Discovery studio 3.5 (Accelerys Inc., USA). For maintaining the structure of the quadruplex, the two internal K^+^ ions were placed between three quartets. The ligand was docked manually between the T1-T2 and G6-T7 steps with orientations obtained from NOE experimental data. A set of NOE distances was introduced as restraints with a force constant of −30 kcal/mol/Å^2^. The drug-quadruplex system was typed in charmM forcefield^51^ followed by solvation using an explicit solvent model. A periodic TIP3P^52^ orthorhombic water box extending to a distance of 10 Å surrounds the complex and contained 679 water molecules. Subsequently, the complex was minimised by 500 steps each of Steepest Descent and Conjugate Gradient algorithms with an RMS gradient of 0.1 and 0.0001, respectively. To obtain the conformations with the lowest potential energy, the quadruplex-ligand complex was subjected to simulated annealing restrained molecular dynamics with the whole set of NOE restraints. Standard dynamic cascade runs were performed on the complex in which the system was heated to 300 K and allowed to equilibrate under constant pressure for 1 ps. The production was done for 1ns in an NPT ensemble where the Hoover constant temperature method specifies the thermal mass with time step of 1 fs. Long range electrostatics were treated with the Particle Mesh Ewald (PME) method^53^, and a 14 Å cut-off radius counted the non-bonded distances. The equation of motion was numerically integrated using the Lepfrog Verlet integrator. To constrain the motion of H-bonds, the SHAKE algorithm^54^ was applied during the whole simulation runs. The coordinates of iterations were saved every 10 steps. The trajectory analysis was performed using Discovery studio client 3.5.

## Additional Information

**Accession code**: The coordinates for NMR model have been deposited in the Protein Data Bank, with accession code 2MS6.

**How to cite this article**: Tawani, A. and Kumar, A. Structural Insight into the interaction of Flavonoids with Human Telomeric Sequence. *Sci. Rep*. **5**, 17574; doi: 10.1038/srep17574 (2015).

## Supplementary Material

Supplementary Information

## Figures and Tables

**Table 1:** The binding constant (K_d_,(M)) values of Quercetin, Rutin and Luteolin with Tel7 G-quadruplex DNA.

**(a) The binding constant (K_d_,(M)) values of Luteolin, Quercetin, Rutin and Genistein with DNA.**			
**Flavonoids**	**Tel7 G-quadruplex DNA**		**ct-DNA**
	**K_d_1(M)**	**K_d_2 (M)**	**K_d_(M)**
Luteolin	2.30 × 10^−8^	4.28 × 10^−5^	2.65 × 10^−5^
Quercetin	4.90 × 10^−9^	4.35 × 10^−6^	4.26 × 10^−6^
Rutin	3.80 × 10^−8^	9.63 × 10^−6^	7.59 × 10^−5^
Genistein	2.65 × 10^−5^	2.65 × 10^−5^	K_d_1 = 2.77 × 10^−7^
			K_d_2 = 41.00
**(b) The binding constant (K_d_,(M)) values of Quercetin with Tel22, c-myc and c-kit21up G-quadruplex DNA.**			
**G-quadurplex DNA**	**Quercetin**		
	K_d_1(M)	K_d_2 (M)	
Tel22	3.02 × 10^−8^	3.40 × 10^−6^	
c-myc	1.38 × 10^−7^	8.33 × 10^−6^	
c-kit21up	1.13 × 10^−6^	–	
