# Supplementary material for: Structural Insight into the interaction of Flavonoids with Human Telomeric Sequence
Source: Sci Rep. 2015 Dec 2;5:17574. doi: 10.1038/srep17574 (PMC4667226; doi:10.1038/srep17574)
Supplement: Supplementary Information [file srep17574-s1.pdf]

Supplementary Information

Structural Insight into the interaction of Flavonoid with Human Telomeric Sequence

Arpita Tawani[1], Amit Kumar[1,]*

[1] Centre for Biosciences and Biomedical Engineering, Indian Institute of Technology Indore, Khandwa Road, Indore, Madhya Pradesh, India

* Corresponding author

Email:amitk@iiti.ac.in (AK)

Index

SI. Supplementary tables

S4-S6

Table S1. Absorption spectral characteristics of Luteolin, Quercetin, Rutin and Genistein with Tel7 G-quadruplex DNA. S4

Table S2a. Life time fluorescence decay parameters for flavonoids and their D/N = 2:1 complex with Tel7 G-quadruplex DNA at 298 K. S4

Table S2b. Life time fluorescence decay parameters for Quercetin and its D/N = 2:1 complex with Tel22 G-quadruplex DNA at 298 K. S4

Table S3. Energy terms (kcal mol^{-1}) for starting structure and final rMD structure. S5

Table S4. Statistics of the solution structure of Quercetin- Tel7 G-quadruplex DNA S5

Table S5. Relative intensity of intermolecular NOE connectivity between Tel7 G-quadruplex DNA and Quercetin in the complex at D/N = 2.0 from NOESY spectra at 298K. S6

SII. Supplementary figures S7-S17

Figure S1. Circular Dichroism titration spectrum for free DNA and in the presence of (a) Luteolin (b) Quercetin (c) Rutin (d) Genistein; at D/N = 2.0 ratio. S7

Figure S2. Circular Dichroism titration spectrum for free Tel22 G-quadruplex DNA and in the presence of Quercetin upto D/N = 2.0 ratio. S8

Figure S3. Absorbance titration curve of (a) Luteolin (b) Quercetin (c) Rutin (d) Genistein; as a function of Tel7 G-quadruplex DNA concentration. S9

Figure S4. Fluorescence titration curve of (a) Luteolin (b) Quercetin (c) Rutin (d) Genistein; as a function of Tel7 G-quadruplex DNA concentration. S10

Figure S5. Fluorescence titration curve of (a) Luteolin (b) Quercetin (c) Rutin (d) Genistein; as a function ct-DNA concentration. S11

Figure S6. Fluorescence titration curve of Quercetin as function of (a) Tel22 (b) c-myc (c) c-kit21up G-quadurplex DNA concentration. S12

Figure S7. Fluorescence lifetime decay curve at 2:1 D/N ratio for Quercetin and Tel22 G-quadurplex DNA. S13

Figure S8. Overlay of one dimensional proton spectra for Quercetin and Tel7 as a function of ligand/DNA ratio upto D/N = 2.0 at 298 K. S14

Figure S9. Expansion of NOESY spectrum showing stacking interaction of G and A-tetrads. S15

Figure S10. Expansion of NOESY spectrum of Tel7 complexed with Quercetin showing loss of sequential connectivity between T1H1´-T2H6 in drug-DNA complex. S16

Figure S11. Interaction of Quercetin at (a)T1/T2 (b) G6/T7 base steps. S17

Figure S12. PCR Stop assay with Tel22. S18

SI. Supplementary Tables

Table S1. Absorption spectral characteristics of Luteolin, Quercetin, Rutin and Genistein with Tel7 G-quadruplex DNA.

Flavonoid	ε_{free} DNA	ε_{bound} Drug	$\Delta\varepsilon$ drug = (ε_{free}- ε_{bound})	λ_{final} (nm)	*$\Delta\lambda$	% Chromicity
**Luteolin**	69800	26488	-6071	359	-11	+28.70
**Quercetin**	69800	16552	-1632	383	-15	+9.67
**Rutin**	69800	23290	-4490	361	-3	+22.15
**Genistein**	69800	43096	-5836	321	+2	+16.60

***$\Delta\lambda = \lambda_{initial} - \lambda_{final}$ [(+) sign shows hypsochromic shift; (-) sign shows bathochromic shift]**
# (+) sign shows hyperchromicity.

Table S2a. Life time fluorescence decay parameters for flavonoids and their D/N = 2:1 complex with Tel7 G-quadruplex DNA at 298 K.

**Flavonoids**	**Uncomplexed Flavonoids**							**Flavonoids and Tel7 complex**						
	**Life Time Decay (ns)**			**Amplitude**				**Life Time Decay (ns)**			**Amplitude**			
	**τ1**	**τ2**	**τ3**	**B1**	**B2**	**B3**	χ^2	**τ1**	**τ2**	**τ3**	**B1**	**B2**	**B3**	χ^2
**Luteolin**	3.2	1.4	7.5	64.4	19.5	16.1	1.36	4.5	14.8	0.16	43.4	21.0	35.6	1.32
**Quercetin**	0.03	2.4	9.3	79.5	7.9	12.5	1.34	3.8	13.1	0.12	34.1	18.0	47.8	1.20
**Rutin**	1.4	2.7	7.5	81.4	9.7	8.9	1.21	4.9	12.8	0.54	64.5	19.3	16.2	1.14
**Genistein**	5.6	21.9	0.5	59.5	29.0	11.5	1.44	5.9	38.3	0.007	0.0	0.0	100.0	1.16

Table S2b. Life time fluorescence decay parameters for Quercetin and its D/N = 2:1 complex with Tel22 G-quadruplex DNA at 298 K.

**Flavonoid**	**Uncomplexed Flavonoids**							**Flavonoids and Tel22 complex**						
	**Life Time Decay (ns)**			**Amplitude**				**Life Time Decay (ns)**			**Amplitude**			
	**τ1**	**τ2**	**τ3**	**B1**	**B2**	**B3**	χ^2	**τ1**	**τ2**	**τ3**	**B1**	**B2**	**B3**	χ^2
**Quercetin**	0.07	0.89	0.07	-1.60	0.02	1.93	1.54	1.09	4.33	0.035	0.007	0.002	0.497	1.19

Table S3. Energy terms (kcal mol^{-1}) for starting structure and final rMD structure.

Structure	Potential energy (kcal mol^{-1})	Van der Waals Energy (kcal mol^{-1})	Electrostatic Energy (kcal mol^{-1})
**Initial**	31204815.569	191.19652	-5659.55869
**Final**	-10256.458	-39.53001	-5596.76652

Table S4. NMR statistics for complexes

**NMR distance**	**Nucleic acid/ligand**
Distance restraints	
Total NOE	712
Intra-residue	336
Inter-residue	96
NOE- derived distance restraints	24
Hydrogen bonds	54
Average pairwise r.m.s.d.** (Å)	
All DNA heavy	2.44
DNA binding site(T1/T2)	2.46
DNA binding site(G6/T7)	2.49
All nucleotides	2.36

**Pairwise r.m.s.d. was calculated among 10 refined structures.

Table S5. Relative intensity of intermolecular NOE connectivity between Tel7 and the Quercetin in the complex at D/N=2.0 from NOESY spectra at 298K.

S.No.	Sequence Connectivity	NOE Intensity	Distances obtained by rMD (Å) (2MS6)
1.	T7H1'-H2'	w	3.80
2.	T7H1'-H6'	ss	2.10
3.	G6H8-H2'	s	2.80
4.	T7H6-H2'	s	2.80
5.	T7H6-H6'	vw	4.50
6.	G6H1-H2'	vw	4.50
7.	G6H1-H6'	ss	2.10
8.	G6H2'1-H8	vw	4.50
9.	G6H2'1-H6	vw	4.50
10.	G7H4'-H8	ss	2.10
11.	G6H3'-H8	ss	2.10
12.	G6H3'-H6	ss	2.10
13.	G6H1-H8	vw	4.50
14.	A3H2-H6	vw	4.50
15.	T1H6-H8	s	2.80
16.	T1H1'-H8	s	2.80
17.	T2H1'-H6	s	2.80
18.	T2H1'-H8	s	2.80
19.	T1H2'2-H8	ss	2.80
20.	T1H2'1-H8	s	2.80
21.	T1H2'1-H2'	s	2.80
22.	T1H2'1-H6'	s	2.80
23.	T1H2'2-H2'	s	2.80
24.	T1H2'2-H6'	s	2.80

SII. Supplementary Figures

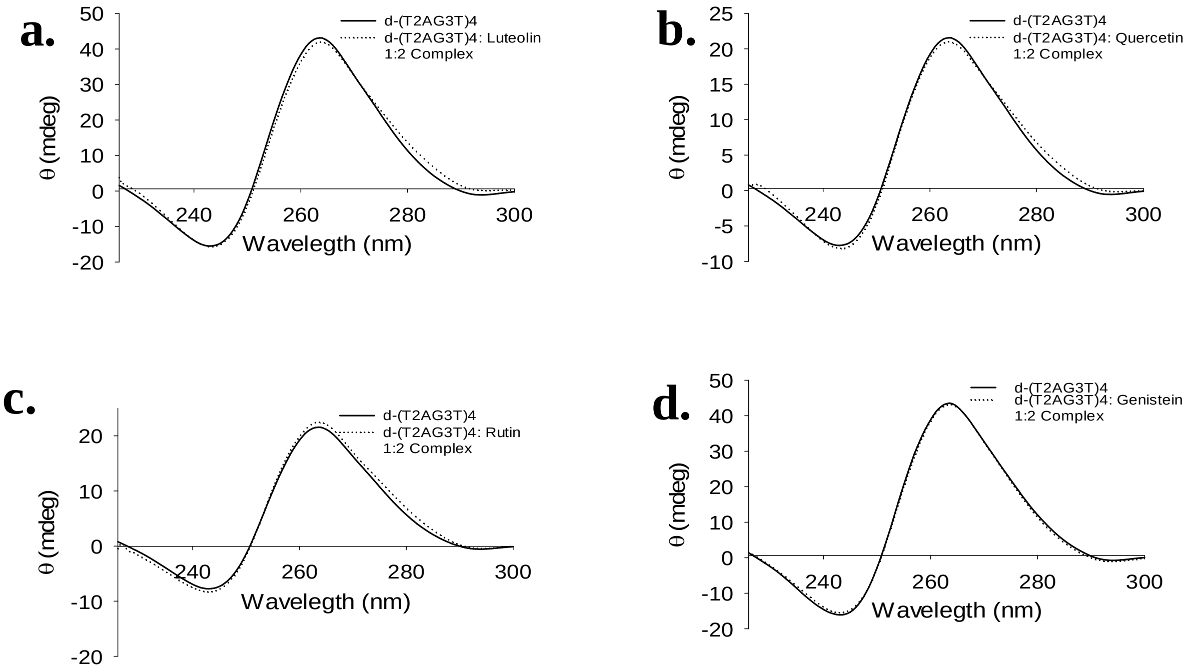

Figure S1. Circular Dichroism titration spectrum for free DNA (straight line) and in the presence of (a) Luteolin (b) Quercetin (c) Rutin (d) Genistein (dotted line); at D/N = 2.0 ratio.

.

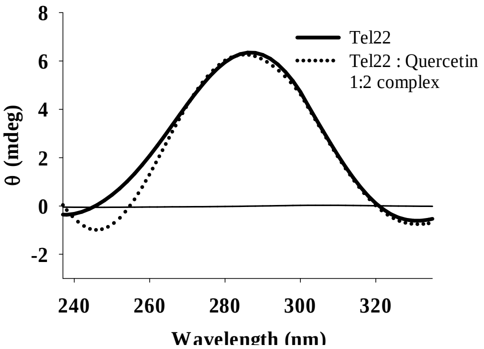

Figure S2. Circular Dichroism titration spectrum for free Tel22 G-quadruplex DNA (straight line) and in the presence of Quercetin (dotted line); at D/N = 2.0 ratio.

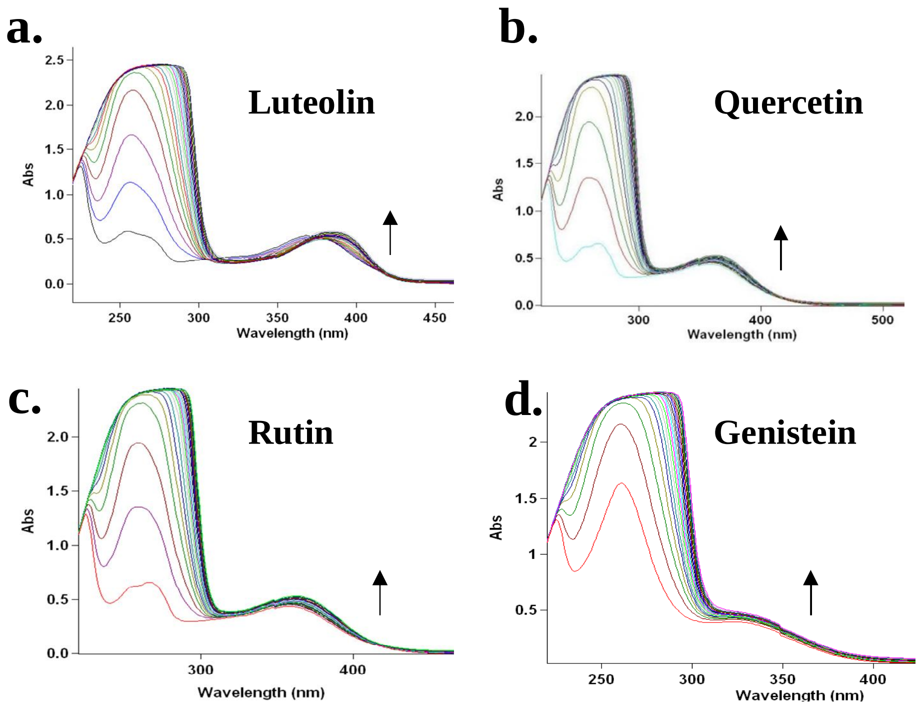

Figure S3. Absorbance titration curve of (a) Luteolin (b) Quercetin (c) Rutin (d) Genistein; as a function Tel7 G-quadruplex DNA concentration.

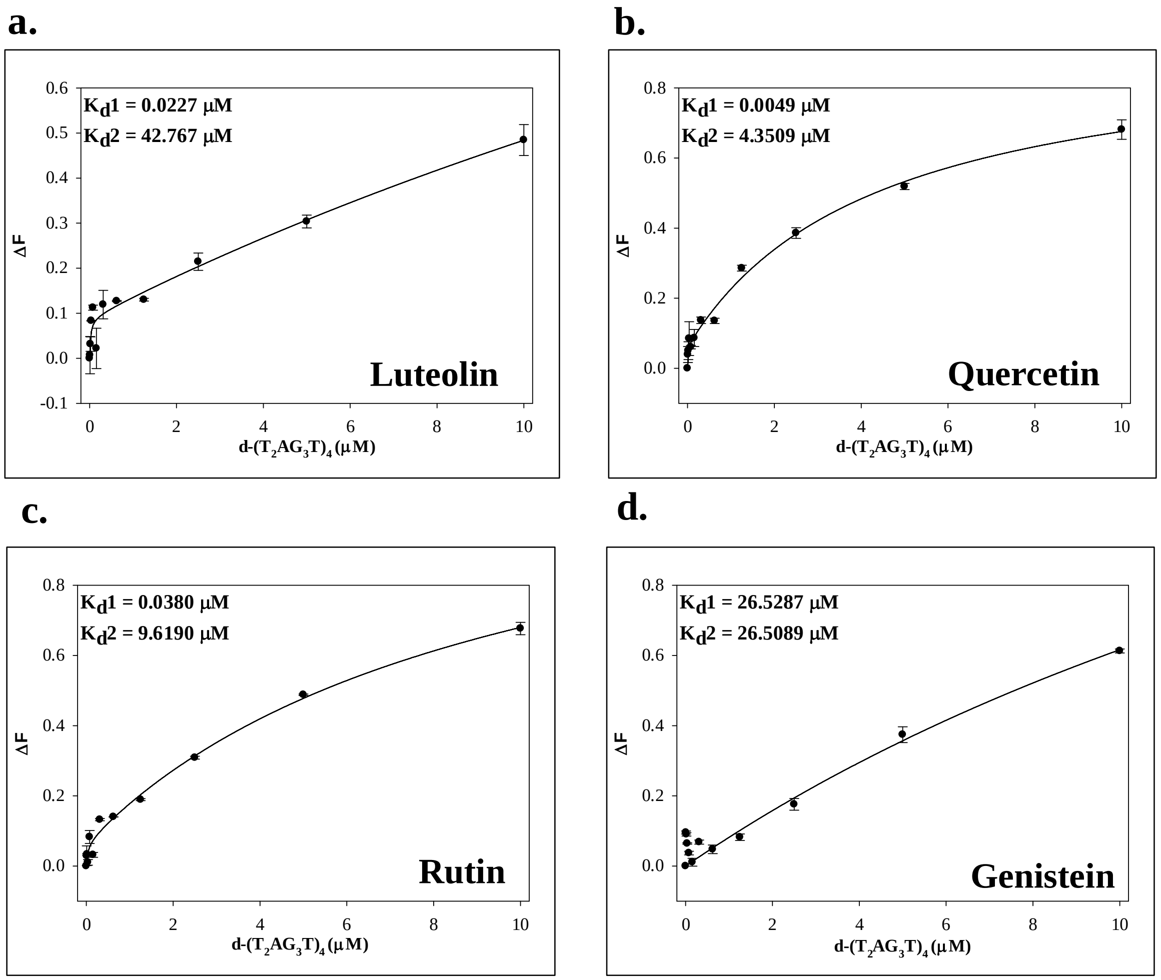

Figure S4. Fluorescence titration curve of (a) Luteolin (b) Quercetin (c) Rutin (d) Genistein; as a function of Tel7 G-quadruplex DNA concentration. Solid lines represent fit according to the ligand binding two site saturation. Value of Binding constant(s) (K_d) are indicated at the top left side of the plot. The normalized data was plotted for Genistein, as it shows quenching.

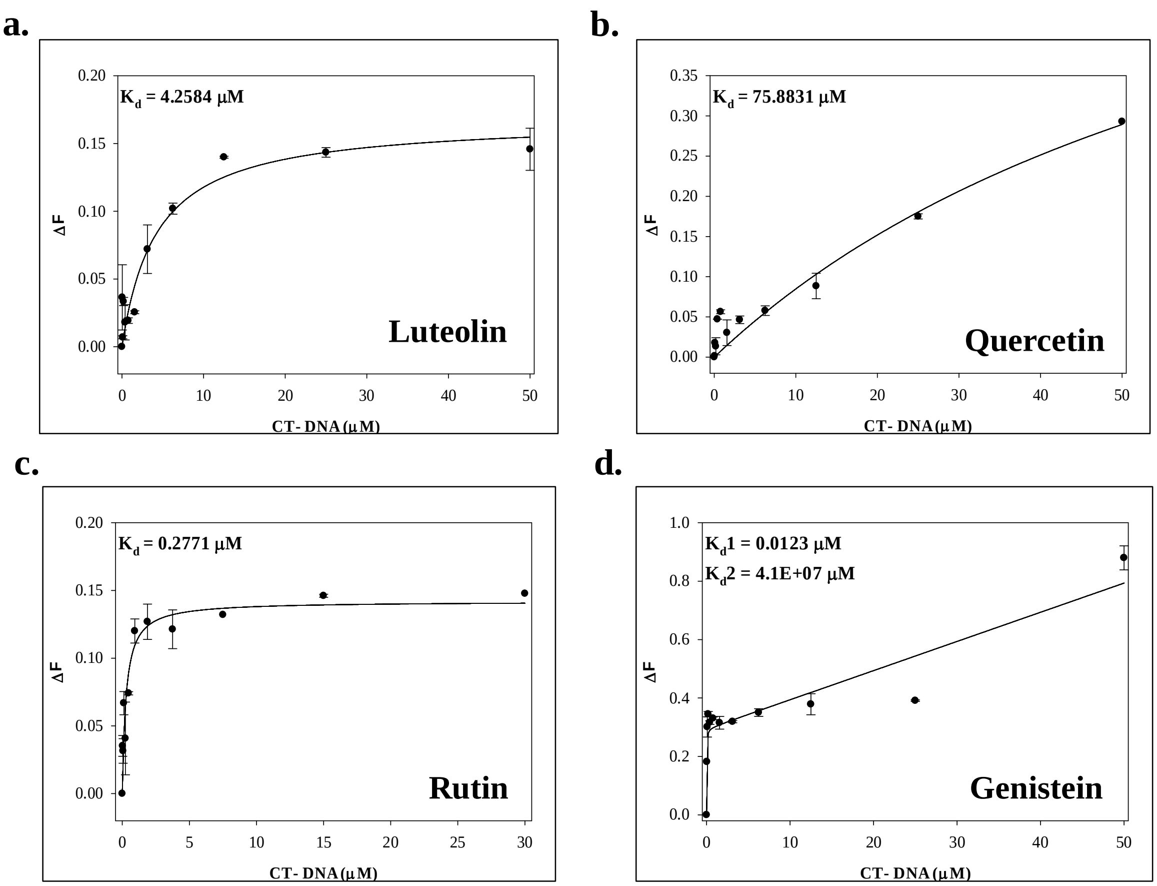

Figure S5. Fluorescence titration curve of (a) Luteolin (b) Quercetin (c) Rutin (d) Genistein; as a function of ct- DNA concentration. Solid lines represent fit according to the ligand binding one site saturation for all except Genistein. Value of Binding constant(s) (K_d) are indicated at the top left side of the plot. The normalized data was plotted for Genistein as it shows quenching.

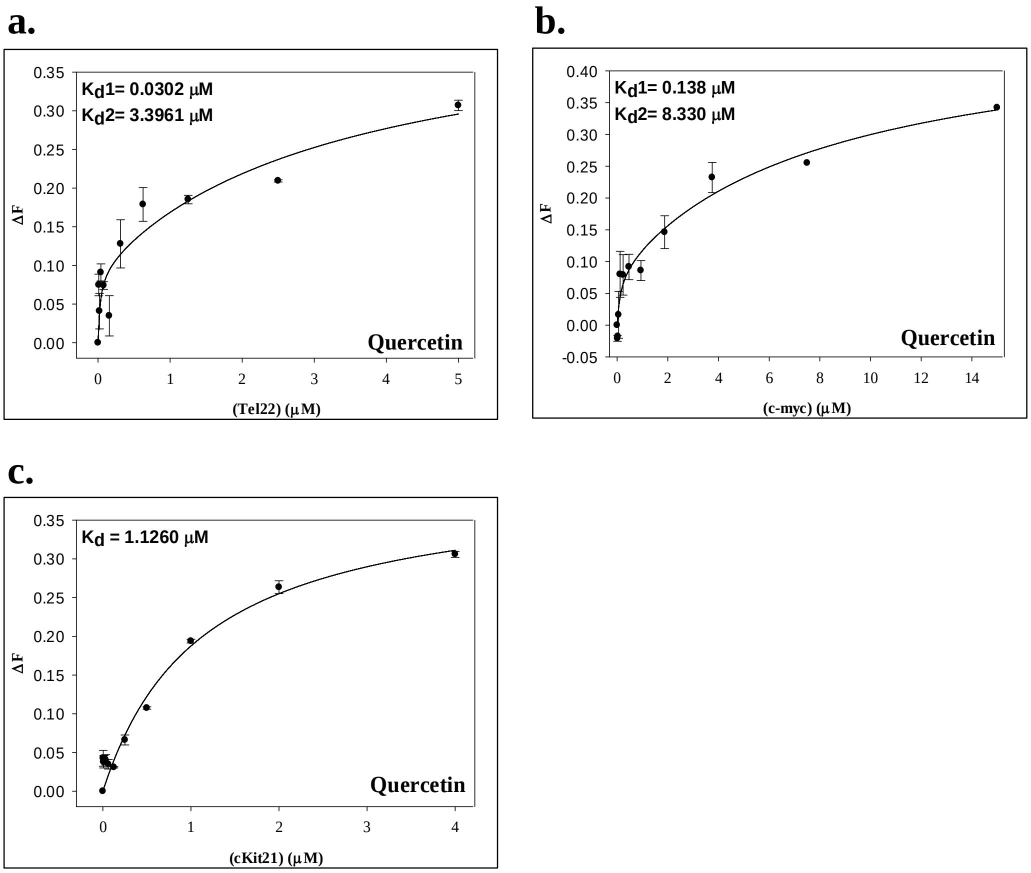

Figure S6. Fluorescence titration curve of Quercetin as function of (a) Tel22 (b) c-myc (c) c-kit21up G-quadurplex DNA concentration. Solid lines represent fit according to the ligand binding two site saturation for all except ckit21up. Value of Binding constant(s) (K_d) are indicated at the top left side of the plot.

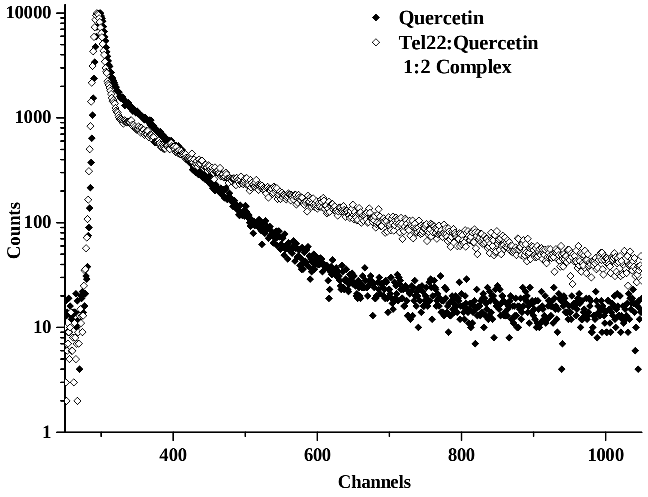

Figure S7. Fluorescence lifetime decay curve at 2:1 D/N ratio for Quercetin and Tel22 G-quadurplex DNA.

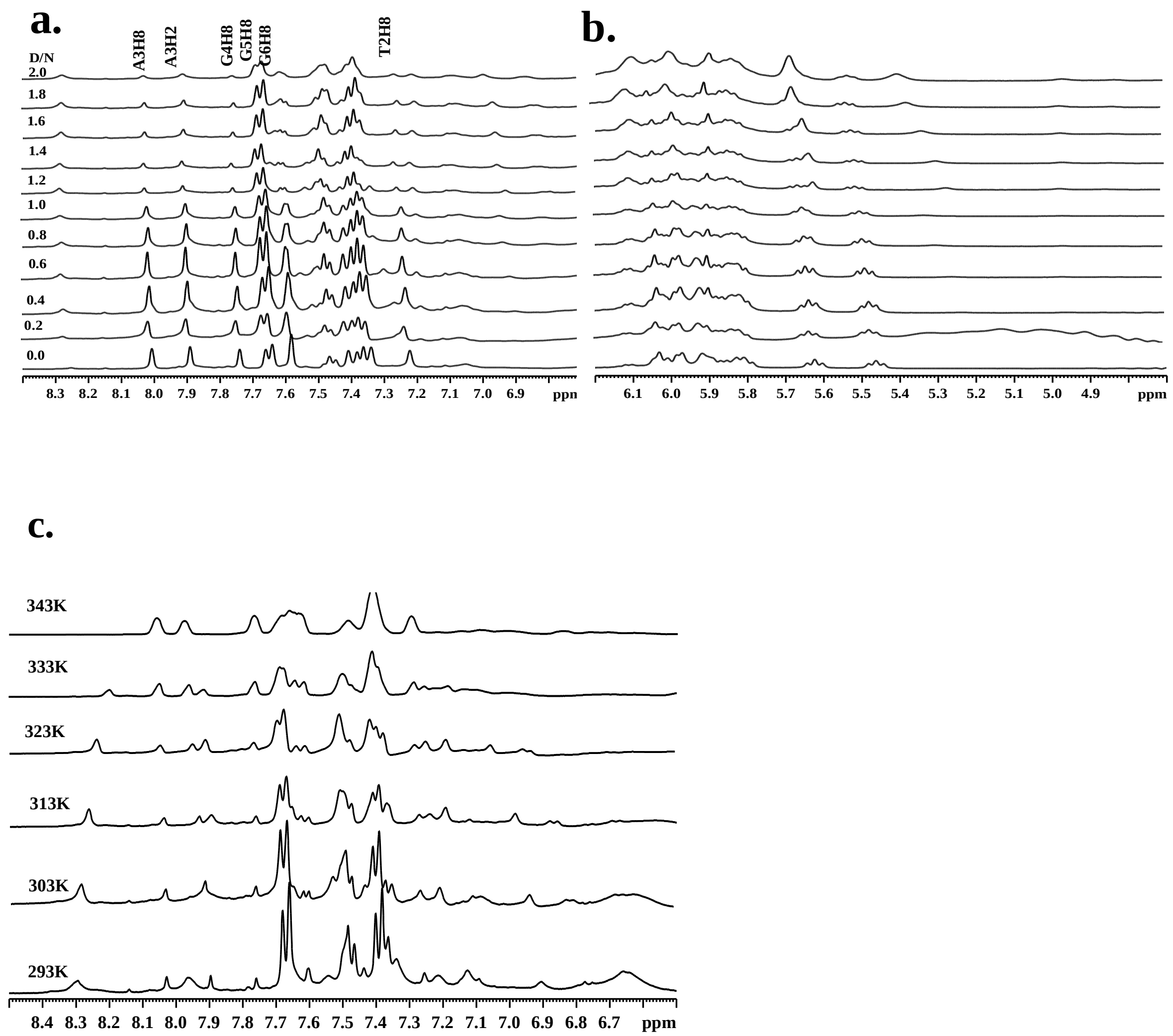

Figure S8. Overlay of one dimensional proton spectra for Quercetin and Tel7 complex. (a) Interaction of Quercetin with Tel7 monitored by base proton region as a function of ligand/DNA ratio upto D/N = 2.0 at 298 K. (b) Interaction of Quercetin with Tel7 monitored by H1' region as a function of ligand/DNA ratio upto D/N = 2.0 at 298 K. (c) Interaction of Quercetin with Tel7 monitored by base proton region as a function of temperature at ligand/DNA ratio = 2.0.

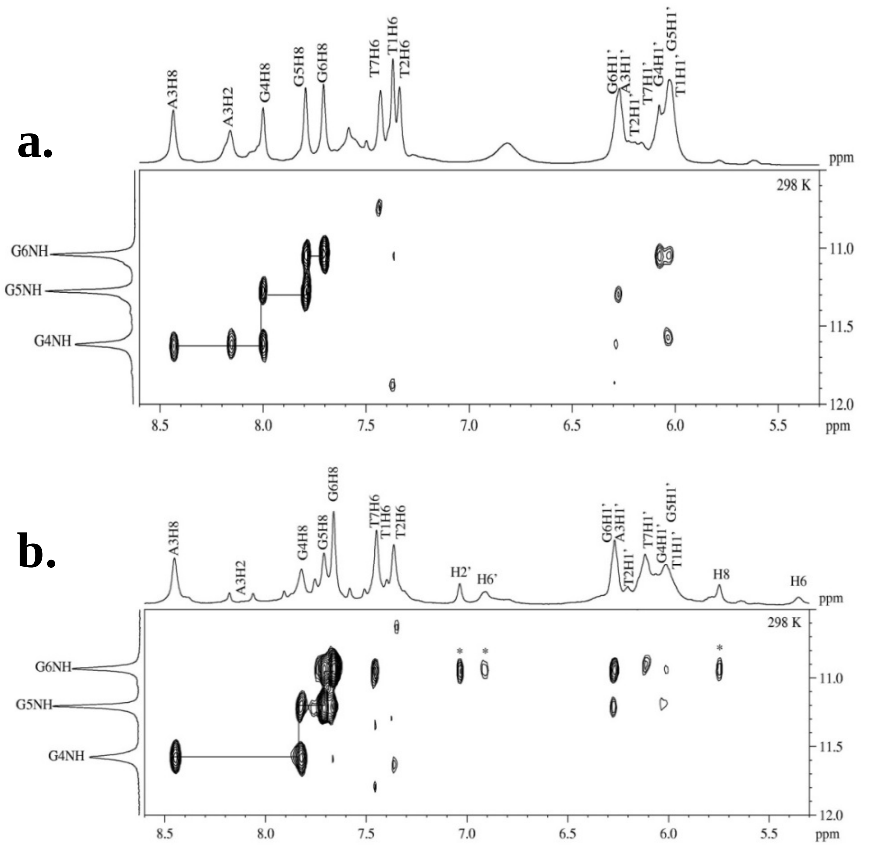

Figure S9. Expansion of NOESY spectrum of (a) unbounded Tel7 showing good stacking interactions of G4NH-A3H2 (b) Quercetin complexed with Tel7 at D/N = 1.0 showing the absence of G4NH-A3H2 NOE and presence of G5NH-G6H8 NOE illustrating perturbations in stacking interaction of G and A-tetrads.

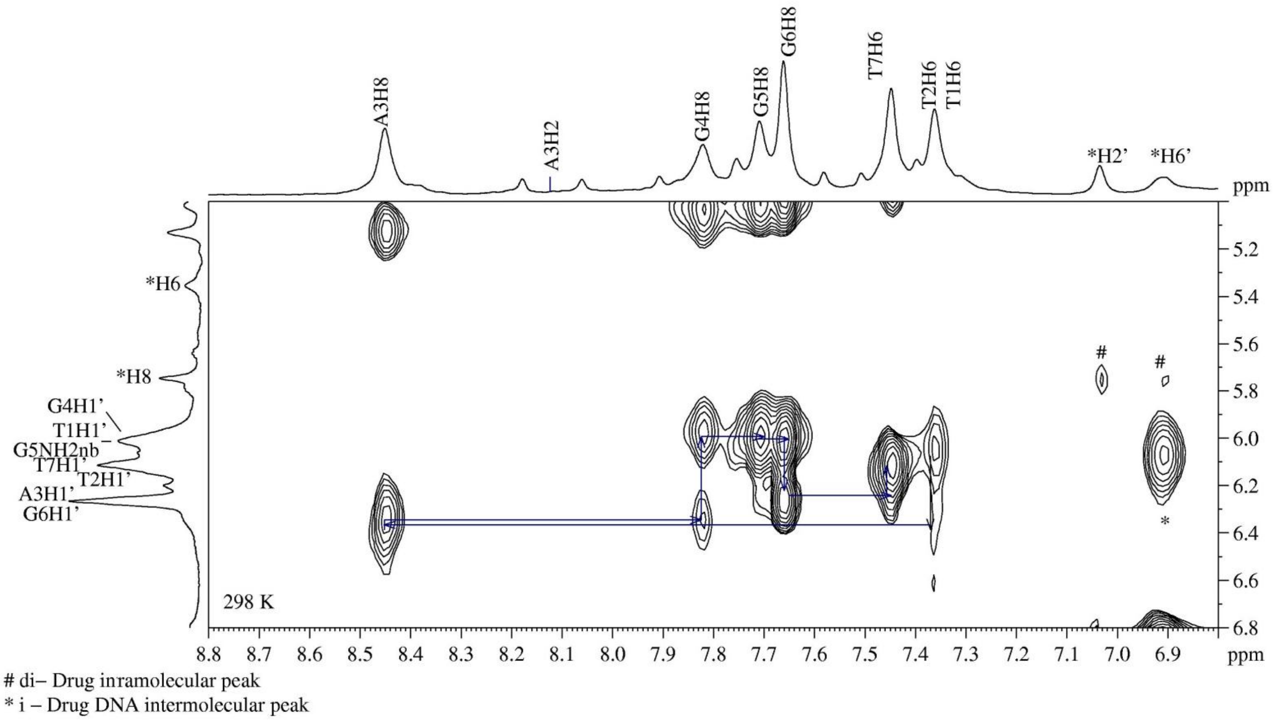

Figure S10. Expansion of NOESY spectrum of Tel7 at D/N = 1.0 complexed with Quercetin showing loss of sequential connectivity between T1H1´-T2H6 in drug-DNA complex.

a. **b.**

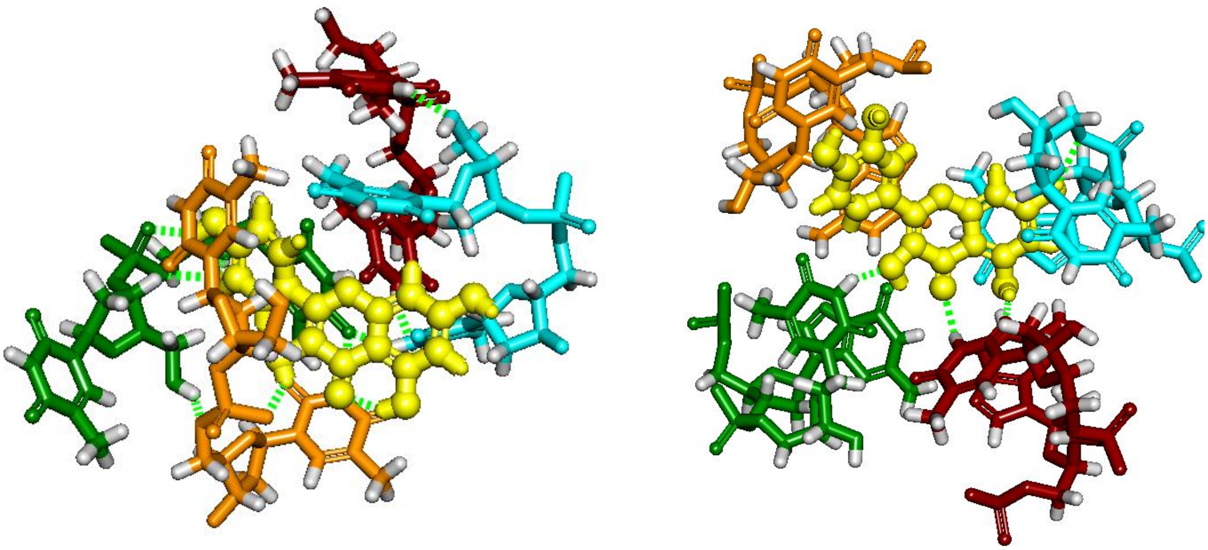

Figure S11. Interaction of Quercetin at (a) T1/T2 (b) G6/T7 base steps. Green dots represent the Hydrogen Bonds.

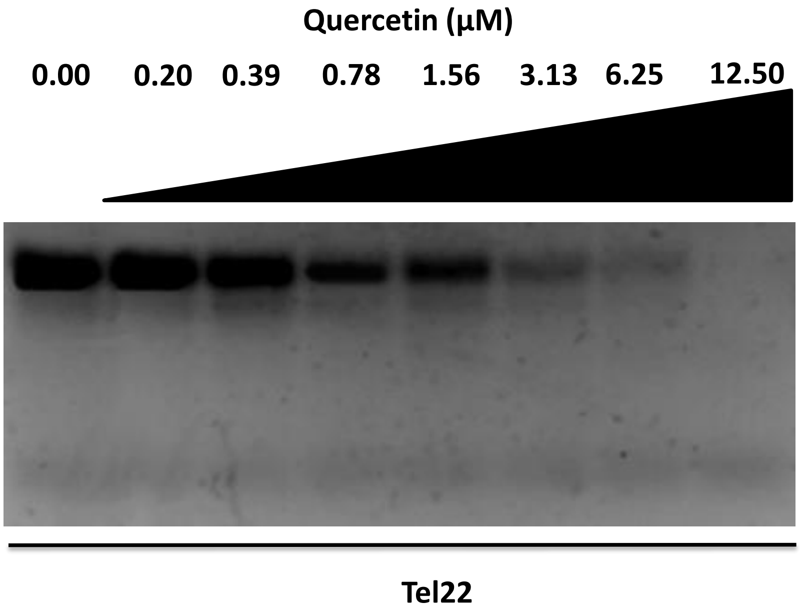

Figure S12. PCR stop assay with Tel22. The assay was performed by employing modified protocol of previous study using a test oligonucleotide Tel22: d-(5'-AGGGTTAGGGTTAGGGTTAGGG-3') and a complementary oligonucleotide (RevTel22): d-(5'-TCTCGTCTTCCCTAA-3'). The observed decrease in the intensity of the PCR product with increasing concentration of Quercetin (lost of band at 12.5 μM) indicates that binding of Quercetin stabilize the G-quadruplex (tel22) and blocks *Taq*polymerase activity of DNA amplification. Assay reactions were performed in a final volume of 25 μL of reaction mixture containing 10mM Tris buffer, 50 mM KCl, 10.0 pmol of each oligonucleotide, 2.5 units of *Taq* polymerase and the amount of the ligand as indicated. Reaction mixtures were incubated in a thermocycler with the following cycling conditions: 94°C for 2 min, followed by 30 cycles of 94°C for 30 s, 58°C for 30 s, and 72°C for 30 s. Amplified products were resolved on a 3% agarose gel in 1XTBE and stained with EtBr. Gel Image was analysed on ImageQuant LAS 4000 (GE Healthcare).
